# Cultural, social, and economic influences on academic field choice among Jewish and Muslim students

**DOI:** 10.1371/journal.pone.0315276

**Published:** 2024-12-06

**Authors:** Michael Zouari, Zeev Shtudiner

**Affiliations:** Department of Economics and Business Administration, Ariel University, Ariel, Israel; University of Haifa, ISRAEL

## Abstract

This study investigated the complex cultural, social, and economic factors influencing the choice of academic field among Jewish and Muslim students in Israeli higher education. By employing a mixed-methods approach, combining quantitative surveys and conjoint analysis, this research revealed significant differences between the two groups. Our findings indicate that Muslim students exhibit significantly higher levels of individualism compared to Jewish students, as measured by the individualism scale. They also place greater importance on prior work experience and teacher influence when choosing a field of study. In contrast, Jewish students prioritize earning potential. The findings underscore the need for culturally responsive educational policies and support systems that cater to the diverse needs and aspirations of students from different socio-cultural backgrounds. This study contributes to understanding the multifaceted dynamics shaping educational choices in multicultural societies and highlights the importance of fostering inclusive learning environments. Future research should include additional ethnic groups and explore the long-term impact of educational pathways on social and economic mobility.

## Introduction

Understanding the factors that motivate students to choose specific fields of study is crucial, as these decisions can significantly impact their professional and personal trajectories [1,2]. Academic research in this field aims to identify the influence of social and cultural variables on student decision-making processes and explore how these factors shape group identities and future aspirations.

In this study, we focus specifically on Muslim students within the Arab community in Israel. The Arab community comprises Muslims, Christians, and Druze; however, Muslims constitute the vast majority, accounting for approximately 83.2% of the Arab population [3]. The remaining Arab citizens are either Druze (9.1%) or Christian (7.7%). This demographic distribution justifies our focus on Muslim students, as they represent the largest subgroup within the Arab community. Additionally, cultural factors unique to the Muslim population, such as traditional norms influencing educational and career pathways, are central to our research objectives. All references to ’Muslim students’ pertain specifically to this subgroup unless otherwise specified. By concentrating on Muslim students, we aim to provide a more nuanced understanding of the factors influencing educational choices within the predominant segment of the Arab community in Israel.

In the Israeli context, significant disparities exist in higher education attainment between Jews and Muslims. According to the Israeli Central Bureau of Statistics [4], approximately 33% of Jewish adults hold academic degrees, compared to about 15% of Arab adults. This educational gap is also reflected in higher education participation rates, with Arab students constituting 18.3% of undergraduate students in Israeli academic institutions. These disparities underscore the importance of investigating the factors influencing academic field choices among these groups to address educational inequalities and promote social mobility.

Our research offers a novel perspective by incorporating an in-depth analysis of economic factors, such as earning potential and education costs, alongside cultural and social influences. We employ two complementary research tools—questionnaire analysis and conjoint analysis—to provide a comprehensive picture of the preferences and motivations distinguishing these two groups. In the unique Israeli context, the issue of choosing fields of study is of particular importance, given the diverse demographic and cultural mosaic that characterizes Israeli society. Israel is a dynamic arena where multiple ethnic and cultural identities intersect, as reflected in studies examining the commonalities and differences between Jews and Muslims in various spheres of life [5]. The Israeli reality, where Jews and Muslims live side by side but face different opportunities and life outcomes, raises questions about the educational and occupational choice patterns of the two groups [6,7].

Sharabi’s [5,6] pioneering research sheds light on the profound differences between Jews and Muslims in Israeli society, both culturally and religiously. These studies focused on various aspects of daily life, including work values, personal and family priorities, and normative behavior patterns in each group. The findings revealed substantial differences in the perceptions and lifestyles of the two groups, as reflected in their attitudes toward fundamental concepts such as family, community, leisure, and religion. These differences are rooted in the cultural and ethnic distinctions that shape the identities of the two groups and influence their value systems, expectations, and aspirations.

Considering these findings, it can be indirectly inferred that there is a need to promote dialogue and influence between cultures. Understanding the essential differences in the perceptions and lifestyles of Jews and Muslims in Israel, as revealed by Sharabi’s research, emphasizes the importance of intercultural communication. A deep and respectful encounter between different groups can contribute to mutual understanding and appreciation of each group’s perspectives and foster tolerance and mutual respect. All of these factors are necessary to strengthen Israel’s diverse social fabric and to cope with the challenges inherent in the encounter between multiple cultural identities.

Guterman et al. [8], examined the differences in motivations for choosing an academic field of study between Jewish and Arab students in Israel. Diverse patterns were found in each group, indicating different perceptions of higher education. The researchers found that while Jewish students tended to prefer fields of study perceived as prestigious and promising high economic rewards, Arab students were more influenced by considerations related to contributing to the Arab community and society and aspired to acquire education and skills that would enable them to promote meaningful social change.

Lev Ari and Mula [9] focused on the cultural aspect of intercultural encounters in training Jewish and Arab teachers in academic institutions. They discovered how influence between groups contributes to the development of skills and changes in attitudes and how this influences the shaping of professional identity. Similarly, the research of Gross and Gamal [10] revealed differences in values and identities among Arab adolescent students stemming from their diverse cultural-national backgrounds. These findings highlight the tension between the Israeli and Arab identities in which students live and help them understand future trends in their professional and occupational choices.

Research has shown that the college experience itself, including interactions with diverse peers, shapes students’ motivations and attitudes regarding their career choices [11,12]. Exposure to multicultural environments during higher education can enhance students’ intercultural competence and influence their decisions to work in diverse settings. Sarid et al. [12] found that Arab students in Israel engage in more activities within multicultural contexts than their Jewish counterparts, indicating a higher level of engagement with multicultural education. This engagement may influence their educational choices and future career paths, particularly in fields that involve multicultural interactions.

In various studies, Hager and Jabareen [13] and Arar et al. [14] revealed differences between Jewish and Muslim students in Israel within the education system. Muslim students face many challenges on their path to higher education, tending toward social advancement and community empowerment. In contrast, Jewish students demonstrate an appreciation for intrinsic values and self-fulfillment. Additional studies, such as those by Kaplan et al. [15], Gross [16], and Lev Ari and Mula [9], clarify how differences in perceptions and attitudes toward national identity, academic experiences, and multicultural approaches influence students’ educational and professional decisions. The findings indicate the need for a culturally sensitive educational approach in Israel.

Two other factors that may impact educational choices are risk aversion and levels of individualism. Risk aversion is a key factor that influences decision-making in uncertain situations [17–20]. Students with higher risk aversion may prefer more secure and stable career paths, while those with lower risk aversion might opt for fields with uncertain outcomes but potentially higher rewards [21]. Patnaik et al. [22] found that risk aversion strongly influences students’ decisions, sometimes even outweighing considerations like future earnings or job security.

Individualism and collectivism are critical cultural dimensions influencing decision-making processes [23,24]. In educational choices, individualistic cultures may prioritize personal interests and self-fulfilment, whereas collectivistic cultures emphasize group goals and social harmony [25]. Investigating individualism provides insight into how students from different cultural backgrounds make educational decisions. In the Israeli context, studies such as those by Auyeung and Sands [26] and Arar et al. [14] provide insights into how individualism and collectivism influence educational choices, focusing on cultural values and differences in motivations and priorities between Jewish and Muslim students. Additionally, research by Phinney et al. [27] and Boudarbat [28] indicates the impact of factors such as ethnic identity, family dependence, and future income projections on educational choice decisions.

In this study, we examine how individualism and risk aversion influence the choice of study field among Jewish and Muslim students. Risk aversion influences the extent to which the student is willing to take steps in their chosen field of study, whether to make a conservative and confident decision with minimal risk or to try to take on a higher risk but with the potential for an unpredictable and rewarding outcome. Individualism, which refers to the value of personal importance and independence, can provide students with autonomy and inner strength, enabling them to make independent decisions and navigate their path in the higher education system. Students characterized by high individualism may choose fields that provide a personal and rewarding experience, independent of the public and their immediate environment. The choice to utilize Triandis’s [23] framework of individualism and collectivism is grounded in its extensive application and relevance in cross-cultural studies, particularly in examining decision-making processes. While a critique regarding binary classifications in social sciences is acknowledged [29], Triandis’s model provides a valuable lens for exploring cultural influences on educational choices in the Israeli context.

A study by Tătar et al. [30] found that international students’ academic choices are context-dependent, reflecting the complex interplay between gender, culture, and economic development in their home countries. This finding underscores the importance of considering global cultural factors when examining educational choices [30]. Similar dynamics have been observed in other multicultural societies. For instance, Malik [31] examines the educational choices of Muslim minority students in India and reveals complex cultural influences affecting their academic trajectories. Indian Muslim students often face disadvantages in accessing educational institutions and exhibit lower educational attainment compared to other groups. In contrast, Israeli Muslim students, while exhibiting higher levels of individualism, remain influenced by collective cultural ties. In both contexts, religious and cultural identities significantly shape educational decisions, though in different ways. Malik [31] highlights that Indian Muslim women face a ’double disadvantage’ due to intersecting gender and religious factors, which limit their educational opportunities. Conversely, our study shows that Israeli Muslim students place more emphasis on teacher influence and prior work experience when choosing their field of study compared to Jewish students. Both studies underscore how minority status affects educational choices; however, the Israeli context demonstrates more successful integration into higher education, with Muslim students actively participating in diverse academic fields despite cultural challenges.

Previous studies have examined various factors influencing students’ educational choices, but relatively few have focused on differences between diverse ethnic or demographic groups. For example, Malgwi et al. [32] found that factors such as interest in the subject, perceived personal skills, and earning potential in the field influence the choice of study area among business students, with some gender differences. However, the study did not examine these effects in the context of ethnic differences. Similarly, Quadlin [33] found that educational funding sources, such as loans, family support, and grants, influence field choice but again without considering differences between distinct ethnic or demographic groups. Therefore, there is still a significant research gap in understanding the influence effect between variables such as family background, funding sources, and personal risk propensity on the academic choices of students from different ethnic groups.

Paolillo and Estes [34] examined twelve central factors influencing career choice among accountants, lawyers, engineers, and physicians in the United States. The variables chosen included, among others, parental influence, employment opportunities, social status, earning potential, required years of study, and necessary skills. The research findings provide insights into the various considerations guiding the career selection process among professionals. The study’s methodology and findings can serve as a basis for comparison and expansion in future research examining the factors influencing the choice of study fields among diverse student populations.

This study’s examination of educational choices in a multicultural context resonates with Appadurai’s [35] broader insights about how modernity is experienced differently across cultural settings. Just as Appadurai demonstrates how global cultural flows are actively interpreted and transformed through local lenses, our research reveals how Muslim and Jewish students negotiate their educational choices through distinct cultural frameworks while simultaneously engaging with shared modern institutions. This perspective helps explain the complex dynamic we observe where Muslim students display higher levels of individualism while remaining connected to collective cultural ties—a pattern that reflects what Appadurai terms the ’tension between cultural homogenization and cultural heterogenization’ in contemporary societies. This theoretical framing enhances our understanding of how students from different backgrounds navigate choices within shared educational institutions while maintaining distinct cultural orientations. Furthermore, our approach aligns with Wikan’s [36] concept of ’resonance’ in cross-cultural understanding, particularly in how we interpret the educational choices of different groups. Wikan argues that understanding across cultural boundaries requires going beyond mere words to engage with shared human experiences. This insight is especially relevant to our finding that Muslim students navigate between individualistic educational choices and collective cultural ties. As Wikan suggests, and Appadurai further elaborates, cultural differences should not be seen as insurmountable barriers but rather as different ways of expressing fundamentally shared human experiences and aspirations. This theoretical perspective helps explain how Jewish and Muslim students can maintain distinct cultural identities while participating in the same educational system and making choices that sometimes transcend traditional cultural boundaries. The concept of resonance is particularly useful in understanding how students from different backgrounds can recognize and relate to each other’s educational motivations and challenges, even while making different choices shaped by their respective cultural contexts.

In the present study, two diverse research tools provided in-depth insights and led to accurate results. In the first stage, a questionnaire was used to gather quantitative information, including demographic, cultural, individualism, risk aversion, national belonging, and religious affiliation variables. This tool allows for understanding the variety of considerations influencing the choice of study field among Jews and Muslims. In the second stage, we used Conjoint Analysis to identify the differences in the importance of these factors, especially in contexts where they were found to be significant in the first stage. Conjoint Analysis deepens the understanding of the relative weight of each choice factor in students’ decisions. The results of the two research tools reinforce each other, enhancing the credibility of the findings and providing more robust support for the research conclusions.

The research findings point to a complex continuum of influences affected by various factors. For example, the frequency of religious practice, such as frequent prayers, negatively influenced the impact of teachers and educators in choosing the field of study. This finding indicates a tendency for students to prefer spiritual trends and principles, which reduces the weight given to external advice. In further examination, Jewish students prioritized fields with higher economic returns. At the same time, their Muslim counterparts appeared to consider additional factors, such as past work experience.

Moreover, the data indicates a strong negative correlation between parents’ socio-economic status and the importance attributed to education costs, particularly highlighting the economic barriers faced by students from low-income backgrounds. Finally, previous work experience was a significant decisive factor for Muslim students due to a cultural emphasis on practical life skills, while it had less influence among Jewish students, who often prioritize academic achievements. These findings indicate significant cultural and socio-economic influences on the students’ educational decision-making process.

By analyzing these differences, this research presents how cultural, economic, and individual factors work together to shape educational choices, providing deep insights for those involved in the field. The study aims to foster an academic infrastructure that is more sensitive to the diverse needs and challenges of students from different backgrounds to contribute to a more united and equitable society. These understandings can serve as a model for other multicultural societies and help improve the status of minority groups, setting a standard that can be a model for replication worldwide. This study also sheds light on a conflict experienced by young Muslims in Israel—the desire to embrace Western culture and individualistic values while still being tied to their family and the collectivistic society in their decision-making processes. This internal struggle reflects the complex cultural dynamics at play and emphasizes the importance of understanding the challenges faced by minority groups in multicultural societies.

## Study 1—Investigating the factors influencing choice of field of study among Jewish and Muslim students through questionnaire analysis

### Method

#### Tools

This study included several stages, described below, aimed at thoroughly investigating and understanding the factors influencing the choice of fields of study among Jewish and Muslim students. The research has been conducted in accordance with the ethical standards outlined by the ethics committee for non-clinical research involving human subjects, Ariel University (approval no. AU-SOC-ZS- 20230418). The first tool used was a quantitative survey designed to collect basic information on various factors related to the choice of field of study. The survey included different questionnaires, each focusing on a different factor relevant to the research. The first part of the questionnaire focused on measuring individualism, according to Triandis et al.’s [23] research, while the second part dealt with measuring factors influencing the choice of field of study, according to Paolillo and Estes [34]. In addition, a third part examined the individual’s attitude toward risk [37], and a fourth part was a socio-demographic questionnaire, including data such as gender, age, marital status, religious affiliation, and more. Each of these parts was intended to provide insights into the social, economic, and cultural dynamics influencing students’ decisions. The participants provided written informed consent.

As part of the first study, reliability testing of the variables was performed using Cronbach’s alpha. For the variable measuring the level of individualism, which was examined using a 12-item questionnaire from Triandis et al.’s research [23], high reliability was found, with an alpha of 0.811. This variable examined how participants viewed themselves, the group, and others and focused on topics such as competitiveness and independence. Regarding the variable measuring risk aversion, which originally consisted of 6 items [37], after removing three items that impaired reliability, the overall reliability of the variable had an alpha of 0.809. This variable examined the tendency to take risks and deal with uncertainty. Finally, the reliability of the variable measuring the choice of field of study according to Paolillo and Estes [34] was also measured, with an alpha of 0.812, reflecting the level of importance participants attribute to the following 12 factors: parental influence, influence of teachers/educators, influence of friends, networking with others in the field, number of job openings in the field, prestige and social status, earning potential (salary), cost of education, years required to complete the field of study, job satisfaction, talent (aptitude) in the field of study, and previous work experience. The high reliability of these variables provides a reliable and stable data basis for further analysis in the study.

In the first study, an electronic survey was sent to all undergraduate students at a particular college. After two weeks, the data were collected, and a comprehensive statistical analysis was conducted using SPSS software. The analysis included examination and processing of the responses, aiming to deepen the understanding of the factors influencing the students’ choice of field of study.

The distribution of socio-demographic variables was examined in the initial stage of the study’s statistical analysis. First, an analysis of the frequency and relative frequency of the qualitative variables was conducted using the chi-square test to identify whether there were statistically significant differences between different groups within the sample. Next, the quantitative variables were examined. First, their distribution was checked using the Kolmogorov-Smirnov test, and when the variables were not normally distributed, the non-parametric Mann-Whitney U test was used to assess the differences between the groups. This lack of a normal distribution led to the use of the Mann-Whitney U test to examine the differences between the groups, allowing for more accurate quantitative data analysis.

#### Sample

The study was conducted at Netanya Academic College, a private institution located in Netanya, Israel. The college offers undergraduate and graduate programs in fields such as Business Administration, Law, Computer Science, Communication, Banking and Finance, and Health Systems Management. The student population is diverse, comprising both Jewish and Arab students. While specific demographic percentages are not publicly disclosed, the college is recognized for its commitment to inclusivity and diversity within the student body. This setting provides valuable insights into the educational choices of students from different cultural backgrounds. However, due to potential differences in demographics and institutional characteristics, the sample may not fully represent all academic institutions in Israel.

The study included 251 participants, 55% of whom were Jewish and approximately 36% of whom were Muslim. The remaining participants were Druze and Christian. Since the study focused on the differences between Jews and Muslims, participants who did not belong to one of these groups were removed from the analysis. After filtering, 229 participants remained. The recruitment period spanned from April 23, 2023, to May 17, 2023.

Table 1 describes the socio-demographic variables of the 229 participants. As shown in the table, significant differences were found in marital status, with a higher percentage of single individuals among Muslims (91.2%), more financial assistance provided to Jews (50.7%), and significant differences were found in faculty choices, employment status, and income levels. Income levels are reported as monthly income in Israeli New Shekels (ILS). The data also revealed notable changes in religious practices, reflecting the existing social-cultural dynamics. These findings contribute to understanding the factors influencing the choice of field of study and point to possible avenues for further investigation and analysis of the relationships among demographic variables, individualism, and risk preferences.

**Table 1:** Description of qualitative socio-demographic variables.

Variable	Total Sample	Jews	Muslims	Significance (Jews/Muslims)
Respondents	229	138 (55.0%)	91 (36.3%)	
**Gender**				
Women	132 (57.64%)	74 (53.6%)	58 (63.7%)	0.13
Men	97 (42.36%)	64 (46.4%)	33 (36.3%)	
**Marital Status**				
Single	181 (79.04%)	98 (71.0%)	83 (91.2%)	<0.001
Married	40 (17.46%)	32 (23.2%)	8 (8.8%)	
Divorced	8 (3.5%)	8 (5.8%)	0 (0.0%)	
**Receiving Financial Assistance/Scholarships**				
No	144 (62.88%)	68 (49.3%)	76 (83.5%)	<0.001
Yes	85 (37.12%)	70 (50.7%)	15 (16.5%)	
**Receiving Advice on Field of Study/Career**				
No	136 (59.39%)	82 (59.4%)	54 (59.3%)	0.99
Yes	93 (40.61%)	56 (40.6%)	37 (40.7%)	
**Faculty**				
Health Systems Management	62 (27.68%)	17 (12.7%)	45 (50.0%)	
Insurance	59 (26.34%)	47 (35.1%)	12 (13.3%)	<0.001
Law	30 (13.39%)	24 (17.9%)	6 (6.7%)	
Business Administration	24 (10.71%)	11 (8.0%)	13 (14.4%)	
Other	49 (21.88%)	35 (26.1%)	14 (15.6%)	
**Socioeconomic Status of Your Parents?**				
Low	28 (12.79%)	18 (13.6%)	10 (11.5%)	0.068
Medium	104 (47.49%)	60 (45.5%)	44 (50.6%)	
High	60 (27.4%)	42 (31.8%)	18 (20.7%)	
Other	27 (12.33%)	12 (9.1%)	15 (17.2%)	
**Your Current Employment Status?**				
Unemployed	29 (12.66%)	12 (8.7%)	17 (18.7%)	<0.001
Part-time	98 (42.79%)	59 (42.8%)	39 (42.9%)	
Full-time	70 (30.57%)	55 (39.9%)	15 (16.5%)	
Other	32 (13.97%)	12 (8.7%)	10 (22.0%)	
**Total Net Household Income**				
Up to 2,500	37 (16.74%)	15 (11.2%)	22 (25.3%)	<0.001
Between 2,501 and 4,000	30 (13.57%)	12 (9.0%)	18 (20.7%)	
Between 4,001 and 5,000	20 (9.05%)	10 (7.5%)	10 (11.5%)	
Between 5,001 and 6,500	14 (6.33%)	6 (4.5%)	8 (9.2%)	
Between 6,501 and 8,000	24 (10.86%)	16 (11.9%)	8 (9.2%)	
Between 8,001 and 10,000	22 (9.95%)	17 (12.7%)	5 (5.7%)	
Between 10,001 and 13,000	17 (7.69%)	14 (10.4%)	3 (3.4%)	
Between 13,001 and 17,000	20 (9.05%)	15 (11.2%)	5 (5.7%)	
Between 17,001 and 24,000	13 (5.88%)	12 (9.0%)	1 (1.1%)	
24,001 and above	24 (10.86%)	17 (12.7%)	17 (12.7%)	
**How often do you pray?**				
Several times a day	69 (30.53%)	13 (9.4%)	56 (63.6%)	<0.001
Once a day	40 (17.70%)	37 (26.8%)	3 (3.4%)	
More than once a week	19 (8.41%)	8 (5.8%)	11 (12.5%)	
Once a week	14 (6.19%)	11 (8.0%)	3 (3.4%)	
Once or three times a month	12 (5.31%)	10 (7.2%)	2 (2.3%)	
A few times a year	27 (11.95%)	24 (17.4%)	3 (3.4%)	
Less frequently	12 (5.31%)	11 (8.0%)	1 (1.1%)	
Never	33 (14.60%)	24 (17.4%)	9 (10.2%)	
**How often do you pray?**				
Never	33 (14.60%)	24 (17.4%)	9 (10.2%)	<0.001
Less frequently	12 (5.31%)	11 (8.0%)	1 (1.1%)	
A few times a year	27 (11.95%)	24 (17.4%)	3 (3.4%)	
Once or three times a month	12 (5.31%)	10 (7.2%)	2 (2.3%)	
Once a week	14 (6.19%)	11 (8.0%)	3 (3.4%)	
More than once a week	19 (8.41%)	8 (5.8%)	11 (12.5%)	
Once a day	40 (17.70%)	37 (26.8%)	3 (3.4%)	
Several times a day	69 (30.53%)	13 (9.4%)	56 (63.6%)	

Note: Statistical significance was determined using the chi-square test. Income levels represent monthly income in Israeli New Shekels (ILS).

These differences in average age and marital status between Jewish and Muslim students can be attributed to several factors. One significant factor is the mandatory military service for Jewish Israelis, which typically lasts for two to three years for men and around two years for women after high school, delaying the start of higher education for Jewish students. In contrast, Muslim citizens of Israel are generally exempt from military service and often begin their university studies at a younger age. Additionally, cultural differences regarding the age of marriage and societal expectations may contribute to variations in marital status between the groups. Sabbah-Karkaby and Stier [38] found that in the Arab society, there remains a strong cultural preference for early marriage, especially among women. Their study indicates that a significant proportion of Muslim women in Israel marry at relatively young ages, with traditional norms and family-based decision making strongly influencing both marriage timing and educational pathways. While educational attainment among Arab women has increased significantly over time, traditional patterns regarding marital behavior continue to shape when and how women pursue their education.

Table 2 presents an overview of the quantitative socio-demographic variables and reveals striking and significant differences between Jewish and Muslim respondents in the sample. A statistically significant difference was found in age, with Jews being older than Muslims. Regarding the connection to the state, Jewish students reported a stronger sense of connection than Muslim students. Among Muslims, a higher sense of belonging to the minority group was recorded. Additionally, Jewish students feel a deeper connection to the country in which they live. These findings emphasize the diversity in the perceptions and experiences of the different groups regarding their religion, identity, and relationships with the state. These implications may be significant for understanding the social and cultural dynamics between populations.

**Table 2:** Description of quantitative socio-demographic variables.

Variable	Total Sample		Jews		Muslims		Sig.
	Mean (Std)	Median	Mean (Std)	Median	Mean (Std)	Median	Statistical Test*
Age	25.83 (8.62)	23	28.53 (9.52)	25	21.73 (4.68)	21	<0.001
To what extent are you satisfied with your available financial resources?	3.85 (1.77)	4	3.94 (1.74)	4	3.70 (1.81)	4	0.333
How often do you think about topics related to religion?	3.43 (1.29)	4	3.58 (1.27)	4	3.21 (1.30)	3	0.030
To what extent do you believe that there is a God or something divine?	4.36 (1.11)	5	4.34 (1.14)	5	4.40 (1.05)	5	0.820
To what extent do you feel connected to the country in which you live?	4.06 (1.10)	4	4.38 (0.92)	5	3.57 (1.18)	4	<0.001
To what extent do you feel that you are part of a minority population?	2.56 (1.41)	2	1.95 (1.20)	1	3.49 (1.16)	3	<0.001
To what extent do you feel that you are treated equally compared to the majority population in your country?	3.51 (1.18)	4	3.67 (1.17)	4	3.27 (1.17)	3	0.011

Note: The variables were not normally distributed. Statistical significance was determined using the Mann-Whitney U test.

In assessing the "individualism" variable, which includes 12 items (see S1 Appendix), Cronbach’s alpha analysis was performed to examine the reliability level of the index. The initial result indicates an alpha value of 0.811, which indicates high reliability and a robust and reliable individualism index. This validates the claim that the individualism index is reliable.

In assessing the "risk aversion" variable, which includes six items (see S1 Appendix), Cronbach’s alpha indicated low reliability. This situation was likely caused by distortions created by reverse questions in the measurement tool, which created ambiguity among respondents. After removing the reverse questions with a low correlation, the Cronbach’s alpha increased to 0.809, indicating a significant improvement in the reliability of the index. After removing the questions, the results indicate a reliable index for measuring risk aversion.

Table 3 describes the differences between Jewish and Muslim respondents regarding individualism and risk aversion. A statistically significant difference was observed in terms of individualism: Muslim students reported higher levels of individualism than Jewish students. In contrast, no significant differences were found between the groups regarding risk aversion.

**Table 3:** Description of differences in individualism and risk aversion between Jews and Muslims.

Research Variables	Reliability	Total Sample		Jews		Muslims		Sig.
	Cronbach’s alpha	Mean (Std)	Median	Mean (Std)	Median	Mean (Std)	Median	Statistical Test*
Individualism	0.811	3.37 (0.71)	3.42	3.22 (0.68)	3.21	3.59 (0.70)	3.59	<0.001
Risk Aversion	0.809	3.57 (1.21)	3.67	3.58 (1.16)	3.67	3.56 (1.29)	3.67	0.79

Note:

*The variables were not normally distributed

** Statistical significance was determined using the Mann-Whitney U test.

The analysis revealed that Muslim students scored significantly higher on the individualism scale (M = 3.59, SD = 0.70, Median = 3.59) compared to Jewish students (M = 3.22, SD = 0.68, Median = 3.21), with this difference being statistically significant (Mann-Whitney U test, p < 0.001).

### Results

**Fig 1:**
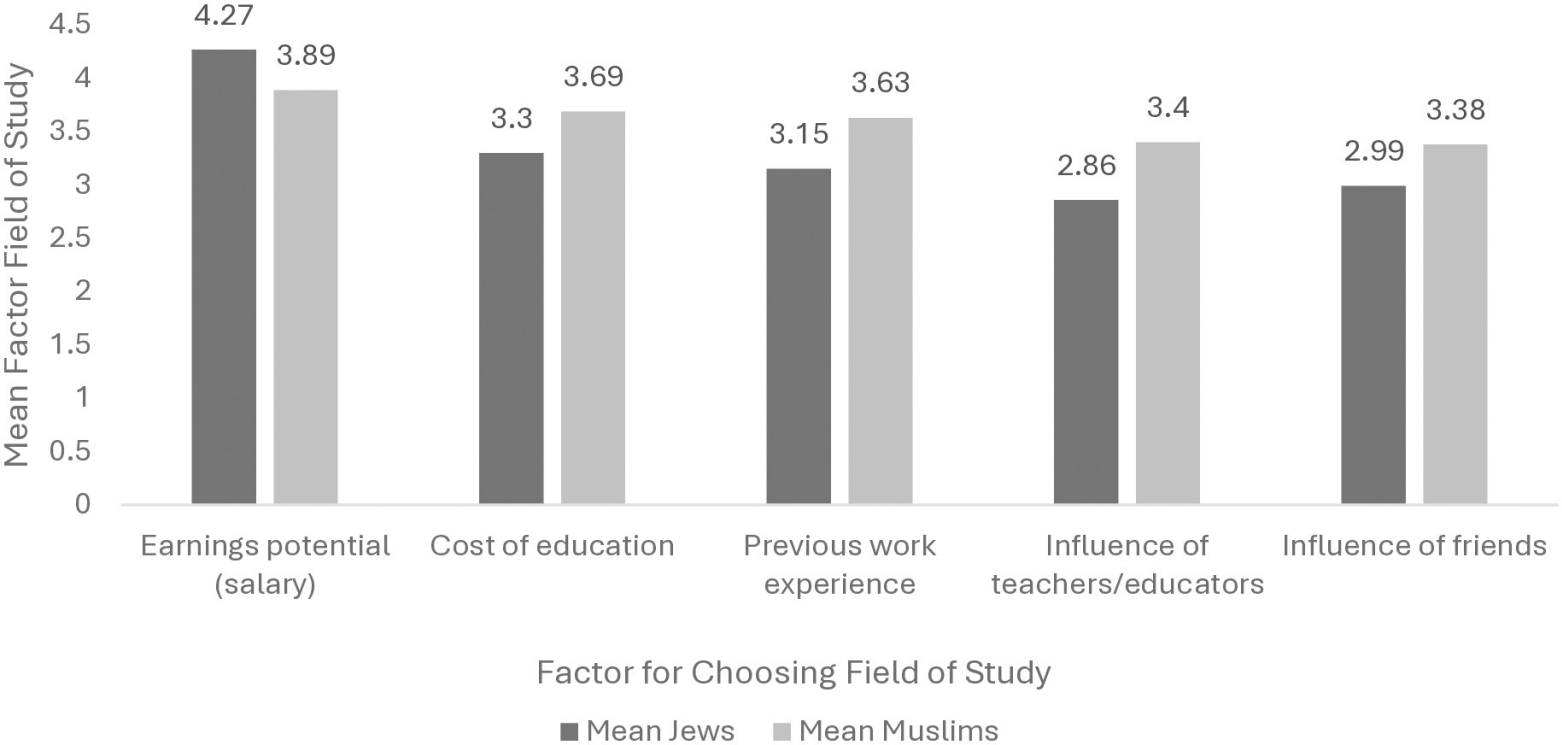
Factors with significant differences for choosing a field of study.

Table 4 provides a detailed description of the factors influencing the choice of field of study between Jewish and Muslim students. From the findings, significant differences were found in several factors, such as the influence of teachers and educators on the choice of field of study being felt more among Muslim respondents, while earning potential is considered a more influential factor among Jews. Additionally, Muslims are more influenced by education costs when choosing a field of study, and they also tend to place more weight on their previous work experience in choosing a field of study. However, the influence of friends on the choice of field of study was found to be higher among Muslims than among Jews. Fig 1 displays the factors with significant differences in choosing a field of study.

**Table 4:** Differences between Jews and Muslims regarding factors for choosing a field of study.

Factors for Choosing a Field of Study	Jews		Muslims		Sig.
	Mean (Std)	Median	Mean (Std)	Median	Statistical Test*
Parental influence	3.78 (1.21)	4	3.88 (1.25)	4	0.394
Influence of teachers/educators	2.86 (1.31)	3	3.40 (1.35)	4	0.003
Influence of friends	2.99 (1.30)	3	3.38 (1.36)	4	0.025
Networking with others in the field	3.86 (1.06)	4	3.97 (0.94)	4	0.600
Number of available positions in the field	3.40 (1.22)	4	3.66 (1.13)	4	0.171
Prestige and social status	3.65 (1.08)	4	3.80 (1.10)	4	0.282
Earnings potential (salary)	4.27 (0.79)	4	3.89 (1.07)	4	0.010
Cost of education	3.30 (1.32)	3	3.69 (1.22)	4	0.032
Years required to complete the field of study	3.54 (1.21)	4	3.51 (1.24)	4	0.872
Job satisfaction	4.18 (0.98)	4	4.03 (1.07)	4	0.331
Talent (aptitude) in the field of study	4.02 (1.06)	4	3.86 (1.11)	4	0.248
Previous work experience	3.15 (1.31)	3	3.63 (1.24)	4	0.010

Note:

*The variables were not normally distributed

** Statistical significance was determined using the Mann-Whitney U test.

These findings reflect different perceptions and expectations between the groups regarding the importance and weight of various factors in choosing a field of study. They may point to broader social and cultural influences.

Hierarchical stepwise regression analysis was conducted as a crucial step in deepening the understanding of the complex dynamics influencing educational decisions. Four separate regressions were run, each with the dependent variable being the rating of a different factor for choosing a field of study: influence of teachers and educators, earning potential, cost of education, and previous work experience. The primary explanatory variable is religious affiliation (Jewish/Muslim), which is examined alongside other explanatory variables such as gender, age, socioeconomic status, frequency of prayers, belonging to a minority group, level of individualism, and risk aversion. This analysis allows for a more detailed and in-depth investigation of the complex and combined influence of various factors on choosing a field of study. Each stage of the hierarchical stepwise regression analysis contributed to the gradual and structured understanding of the interaction between the different variables, aiding in the comprehension of the driving forces behind the educational decision-making process of students in diverse cultural and social environments.

The following are the stages of the hierarchical stepwise regression for examining the factors influencing the choice of field of study:

**Stage 1**: The religious affiliation variable was entered as a dummy variable, with a value of 1 representing Jewish and a value of 0 representing Muslim. This allows us to examine the influence of religion on the choice of field of study.
**Stage 2**: The variables of gender and age were added to account for additional demographic differences.
**Stage 3**: Variables representing the socioeconomic status of the parents and the student’s total net income were added to investigate the relationships between economic factors and the choice of field of study.
**Stage 4**: Variables related to prayer frequency, sense of belonging to a minority group, and level of connection to the country were added to explore social and cultural connections.
**Stage 5**: The central variables of the study, individualism and risk aversion, were entered into the model for an in-depth analysis of their relationships with the choice of field of study.

Table 5 presents the results and thoroughly describes the effects of the various independent variables on the factors for choosing a field of study. It is important to note that the table includes only the variables with coefficients that are significantly different from zero. The comprehensive analysis identifies which independent variables significantly influence each factor and how they affect the student’s decision-making process in choosing a field of study. The extensive analysis conducted in Table 5 forms the core of the research and provides crucial insights into the key factors influencing the students in choosing their field of study.

**Table 5:** Final models presenting the influence of independent variables on different factors for choosing a field of study.

Factors for Choosing a Field of Study	Independent Variable	Standardized Beta Coefficient	Standard Error	t-value	Sig.
**Influence of teachers/educators**	Frequency of prayers	-0.210	0.035	-3.099	0.002
**Earnings potential (salary)**	Jewish/Muslim (dichotomous)	0.238	0.127	3.514	0.001
	Risk aversion	0.165	0.051	2.417	0.017
	Individualism	0.166	0.090	2.365	0.019
**Cost of education**	Parents’ socioeconomic status	-0.148	0.096	-2.199	0.029
	Extent of belonging to a minority group	0.215	0.062	3.197	0.002
	Risk aversion	0.166	0.071	2.511	0.013
**Previous work experience**	Jewish/Muslim (dichotomous)	-0.148	0.183	-2.142	0.033
	Individualism	0.183	0.126	2.648	0.009

Influence of Teachers/Educators–The variable of prayer frequency negatively influences the impact of teachers/educators as a factor in choosing a field of study, indicating a complex relationship between religious practices and educational guidance.

Earning Potential (Salary)–Three independent variables influence earning potential when choosing a field of study. The dichotomous variable of religious identity (Jewish/Muslim) positively influences earning potential. In other words, the influence is more significant for Jewish students. Additionally, the variables "risk aversion" and "individualism" positively influenced earning potential but to a lesser extent than did the dichotomous variable.

Cost of Education–Three independent variables were found to influence the cost of education in choosing a field of study. The parents’ socioeconomic status was found to negatively influence education costs. In contrast, two additional variables–the degree of belonging to a minority group and risk aversion–have a positive influence on the cost of education. Among the three variables, the degree of belonging to a minority group was found to have a more dominant influence on the dependent variable. The findings point to the essential role of socioeconomic and cultural factors in choosing a field of study.

Previous Work Experience–The dichotomous variable Jewish/Muslim was found to negatively influence previous work experience as a decisive factor in choosing a field of study. In contrast, the student’s level of individualism was measured as positively influencing work experience. The findings may indicate cultural directions or trends that influence employment opportunities.

### Conclusions

In the first study, a detailed analysis was conducted on the factors influencing the choice of fields of study among Jewish and Muslim students, focusing on individualism, individual risk preference, and socio-demographic variables. The study revealed significant differences in levels of individualism, with Muslim students exhibiting higher individualism. In contrast, no notable differences were observed in students’ risk preferences, indicating a similar level of risk tolerance in educational choices among both ethnic groups.

Socio-demographic factors, including family status, financial support, and previous work experience, significantly influenced decisions in choosing a field of study. Moreover, Muslim students showed a more significant influence of educators in their choice of field of study, in contrast to the tendency of Jewish students toward fields offering higher earning potential and career opportunities.

These findings emphasize the complex interaction of cultural and social dynamics in educational decision-making, reflecting diverse perspectives and experiences related to religious identity, social connections, and minority status. This research contributes to a deeper understanding of the varied factors influencing educational choices among Jewish and Muslim students and enriches the broader discourse on educational diversity and cultural influences.

This study challenges existing theories that primarily focus on economic and social factors by highlighting the importance of cultural and religious identities and offers a more nuanced approach that incorporates these considerations. The results underscore the need for culturally sensitive educational policies and practices and support the integration of multicultural perspectives in educational guidance, especially in diverse societies. This research provides a strong foundation for future academic inquiry. It is a valuable guide for educational policymakers and practitioners striving to foster more inclusive and culturally aware educational learning environments. The insights from this study encourage ongoing efforts to understand and address the diverse needs of students from different ethnic backgrounds while emphasizing the value of embracing diversity in educational settings.

## Study 2—Investigating the factors influencing the choice of field of study between Jewish and Muslim students using conjoint analysis

### Method

In the second study, a conjoint analysis survey was sent using the tools of "1000minds.com" by the method of Hansen and Ombler [39]. A Conjoint Analysis survey is a research method used primarily to understand consumer preferences by presenting respondents with different combinations of product or service attributes. It has also been applied to study other types of preferences [40,41]. The survey focused on four main factors influencing the choice of field of study, based on the findings from the first study: influence of teachers and educators, earning potential (salary), cost of education, and previous work experience. Each factor was rated as low, medium, or high. The participants provided written informed consent. The recruitment period spanned from June 22, 2023, to July 11, 2023.

Respondents were asked to compare two hypothetical profiles of fields of study, with each profile presenting two out of the four factors, with a specific value for each factor (low, medium, or high). For example, one profile might present "a field highly recommended by teachers/educators" alongside "a field with medium education cost." In contrast, the second profile presents "a field with low recommendation from teachers/educators" and "a field with low education cost." Participants were required to choose their preferred profile from each pair of profiles presented to them.

**Fig 2:**
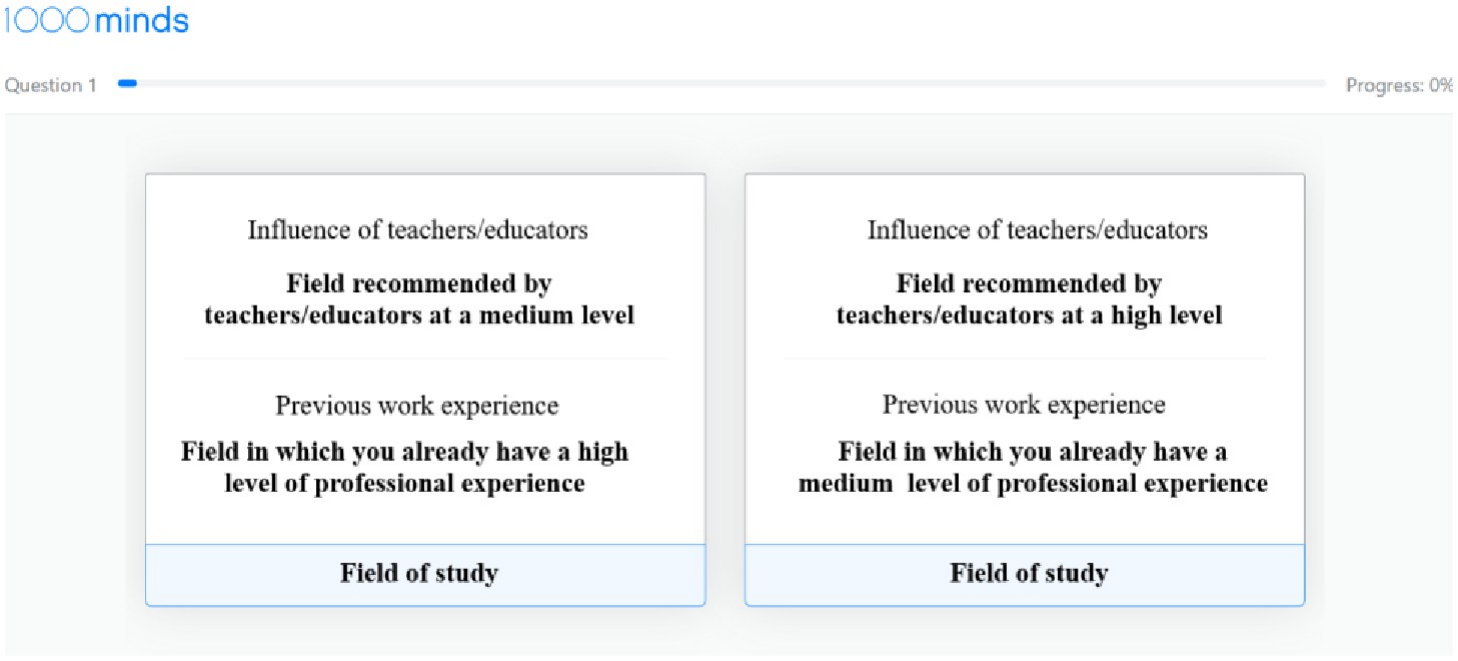
Example of a screenshot for choosing between two profiles of fields of study.

The software presents a series of comparisons between pairs of profiles, each time presenting two different factors with different values (low, medium, or high). The respondent is asked to choose their preferred profile between the two. An example of such a comparison is shown in Fig 2. Based on the respondents’ decisions in all the comparisons, the software calculates the relative weight given to each of the four factors in choosing a field of study for the respondents.

The final output for each participant is the relative percentage of importance they assign to each of the four factors when choosing a field of study, such that the sum of the weights of all factors together equals 100%. Out of approximately 3,000 students approached by the survey, responses were received from 158 participants. After two weeks, all the data were collected, processed, and analyzed using SPSS. The collected data constituted a comprehensive database that allowed for an accurate and in-depth examination of the factors influencing the choice of field of study among Jewish and Muslim students.

First, an analysis of the distribution of qualitative socio-demographic variables was conducted. This analysis included examining the frequency and relative frequency and using the chi-square test to examine the statistical differences between the qualitative variables. Next, the research focused on analyzing the quantitative variables: the Kolmogorov-Smirnov test was performed to examine the distribution of the variables, and when they were found to be non-normally distributed, the data was transferred to the Mann-Whitney U test. This analysis served as a basis for examining the differences between the groups regarding the quantitative variables, providing a solid foundation for the subsequent statistical analyses in the study.

### Results

Out of a total of 158 respondents who answered the conjoint analysis survey, 107 were Jewish (67.7%), while 51 were Muslim (32.3%). Description of socio-demographic variables is displayed in Table 6. The findings of the sample analysis revealed significant differences between Jewish and Muslim students. A significant difference was found in the choice of fields of study (p<0.001), with Jewish students tending more toward the Faculty of Insurance (41.1%) and Behavioral Sciences (14%) than Muslim students who show a higher tendency toward the Faculty of Health System Management (47.1%). Significant differences were found in terms of income levels (p = 0.007), as Jewish students are more evenly distributed across income levels. In contrast, Muslim students exhibit higher percentages in lower income categories, such as 21.6% in the category of up to 2,500 ILS per month. In terms of gender distribution and level of religiosity, no notable differences were found between the two groups, with similar percentages between genders.

**Table 6:** Description of socio-demographic variables.

Variable	Total Sample	Jews	Muslims	Significance (Jews/Muslims)
Respondents	158	107 (67.7%)	51 (32.3%)	
**Gender**				
Women	87 (55.1%)	59 (55.1%)	28 (54.9%)	0.978
Men	71 (44.9%)	48 (44.9%)	23 (45.1%)	
**Faculty**				
Health Systems Management	37 (23.4%)	13 (12.1%)	24 (47.1%)	<0.001
Insurance	56 (35.4%)	44 (41.1%)	12 (23.5%)	
Law	15 (9.5%)	12 (11.2%)	3 (5.9%)	
Business Administration	19 (12.0%)	12 (11.2%)	7 (13.7%)	
Other	31 (19.6%)	26 (24.3%)	5 (9.8%)	
**Level of Religiosity**				
Religious	29 (18.4%)	19 (17.8%)	10 (19.6%)	0.101
Secular	52 (32.9%)	41 (38.3%)	11 (21.6%)	
Traditional	77 (48.7%)	47 (43.9%)	30 (58.8%)	
**Total Net Household Income**				
Up to 2,500	19 (12.3%)	8 (7.6%)	11 (22.0%)	0.007
Between 2,501 and 4,000	23 (14.8%)	12 (11.4%)	11 (22.0%)	
Between 4,001 and 5,000	17 (11.0%)	9 (8.6%)	8 (16.0%)	
Between 5,001 and 6,500	13 (8.4%)	9 (8.6%)	4 (8.0%)	
Between 6,501 and 8,000	18 (11.6%)	12 (11.4%)	6 (12.0%)	
Between 8,001 and 10,000	15 (9.7%)	10 (9.5%)	5 (10.0%)	
Between 10,001 and 13,000	9 (5.8%)	8 (7.6%)	1 (2.0%)	
Between 13,001 and 17,000	14 (9.0%)	12 (11.4%)	2 (4.0%)	
Between 17,001 and 24,000	17 (11.0%)	17 (16.2%)	0 (0.0%)	
24,001 and above	10 (6.5%)	8 (7.6%)	2 (4.0%)	

In the first study, significant differences were found between Jewish and Muslim students in the following factors influencing the choice of field of study: earning potential, work experience, and the influence of teachers and educators. Jewish students gave relatively more significant weight to medium and high earning levels, while Muslims gave relatively more significant weight to high work experience and the influence of teachers and educators. The second study used these factors, and the findings provide a deeper understanding of cultural preferences and influences in choosing a field of study. The findings in Table 7 are similar to and consistent with the results found in the first study and help advance the understanding of the central influences and factors affecting the choice of field of study between different ethnic groups.

**Table 7:** Analysis of differences in factors influencing the choice of field of study between Jews and Muslims.

Factors for Choosing a Field of Study	Jews		Muslims		Sig.
	Mean (Std)	Median	Mean (Std)	Median	Statistical Test
Earnings potential: Medium	19.31% (8.54%)	21.15%	14.57% (8.85%)	11.69%	0.001
Earnings potential: High	38.35% (12.39%)	39.62%	30.55% (12.74%)	29.79%	<0.001
Previous work experience: High	21.55% (10.09%)	21.05%	24.82% (10.15%)	25.46%	0.043
Influence of teachers/educators: High	20.25% (10.58%)	18.64%	23.62% (11.53%)	20.41%	0.093

Note: These values represent the relative importance, or weight, of the attributes and levels within each attribute. The variables were not normally distributed. Statistical significance was determined using the Mann-Whitney U test.

### Conclusions

The application of Conjoint Analysis using the PAPRIKA method and 1000minds software in the second study significantly enhanced our understanding of the factors influencing field of study choices. This approach offered several key advantages: it freed researchers from predetermined profile selections, instead dynamically presenting optimal profiles to respondents based on their previous answers. This adaptive process ensured maximum information extraction with a minimal number of questions, thereby increasing survey efficiency. The method’s use of simple pairwise comparisons, where profiles differ on only two factors, simplified decision-making for respondents, reducing potential biases or errors. Furthermore, the automatic analysis of results through the PAPRIKA algorithm yielded accurate and reliable factor values reflecting respondents’ preferences, eliminating the need for manual data processing and enhancing objectivity.

The approach in the second study allowed us to accurately and profoundly reveal and measure their relative importance while also supporting the findings obtained from the measurement tools in the first study. The second study illuminated the central factors influencing choices of fields of study between Jewish and Muslim students, emphasizing the influence of teachers, earning potential, cost of education, and previous work experience. Jewish students clearly preferred medium to high earning potential in their choice of fields of study. In contrast, Muslim students placed greater weight on high previous work experience and the influence of teachers. The findings highlight complex decision-making processes and the importance of incorporating cultural considerations into educational theories and applications. The findings suggest that educational policies must be tailored to account for these diverse influences, emphasizing the need for culturally coordinated guidance and support systems. Although the study offers valuable insights, its limitations include its focus on only two ethnic groups and the use of self-reported data, which may not capture the full complexity of the decision-making process. Future research should include additional groups and explore additional socioeconomic variables. Comparative studies between ethnic groups and longitudinal studies tracking decision-making processes over time will provide further insights. This research contributes significantly to understanding how cultural, economic, and social factors intersect in shaping educational choices and provides crucial information for decision-makers and educators. This highlights the need for more inclusive and responsive educational frameworks that consider the diverse needs of students from different cultural backgrounds. Ultimately, this research provides a foundation for future efforts to create equitable and effective education systems that recognize and respect the diverse nature of students’ decision-making processes in multicultural societies.

## General discussion

This research investigates the factors influencing the choice of fields of study among Jewish and Muslim students in Israel to uncover the complex cultural, social, and economic influences on these significant decisions. These studies challenge prevailing perceptions of how cultural and social identities shape students’ patterns of thought and action when choosing an academic path. The research findings indicate that factors such as the influence of teachers and educators, earning potential, cost of education, and previous work experience reflect fundamental differences between Jewish and Muslim students rooted in unique cultural and socioeconomic contexts. These insights resonate with the studies of Kaplan et al. [15] and Gross [16], providing a conceptual foundation for understanding the differences in perceptions and attitudes toward national identity and academic experiences among Jewish and Muslim students in Israel and serving as an established starting point for discussing the current findings.

This chapter presents a comprehensive review of the central findings emerging from the two studies conducted, alongside an in-depth discussion of the importance of the topic in the broader socio-cultural context and its contribution to the advancement of knowledge in the field of academic research. The first study was based on a comprehensive quantitative analysis of data collected through surveys, focusing on a wide range of factors with potential influence on the choice of field of study. In contrast, the second study relied on an innovative application of the Conjoint Analysis method to determine the relative importance of those factors. The strategic combination of these two complementary research approaches allows for a more comprehensive and profound picture of the factors influencing students’ educational decisions, recognizing the unique contribution of each approach to enriching the understanding of the cultural and social dynamics that shape students’ academic choice paths. This perception aligns with the groundbreaking works of Sharabi [5,6] and Lev Ari and Mula [9], providing a theoretical framework for understanding the complexity of interactions between culture and education and their impact on decision-making processes.

In an academic era characterized by the intensification of diversity and socio-cultural inclusion trends, it is crucial to devote increased research attention to the diverse influences on educational choices among students from different cultural and religious backgrounds. A deep understanding of the defining factors for these choices forms a solid basis for developing personalized educational policies sensitive to the full range of unique needs and challenges of heterogeneous population groups and aiming to create an inclusive learning space that respects human diversity. This perspective integrates with the insights of Guterman et al. [8], emphasizing the need to adapt the academic system to diverse realities and foster an educational climate that encourages integration and student empowerment.

Within the research framework, findings with high statistical significance were revealed, shedding new light on the fundamental differences between Jewish and Muslim students in their preferred fields of study. The data indicate that while Jewish students focus on fields associated with high earning potential and desirable social prestige, their Muslim peers attach greater importance to choosing fields that allow for meaningful contributions to community life and show a significant affinity for personal areas of interest or previous work experience. These findings echo the insights of Hager and Jabareen [13], pointing to the gap between the two groups regarding social aspirations and self-fulfillment. The second study, which made fruitful use of the advanced tool of Conjoint Analysis, provided quantitative confirmation of the primacy given to particular factors over others and emphasized the fundamental cultural and economic differences that shape educational decision-making processes. These results align with the pioneering research of Arar et al. [14], revealing the decisive influence of cultural and historical baggage on educational attitudes and aspirations among Arab students.

A comprehensive analysis in the first study revealed significant disparities in key demographic characteristics such as marital status, financial support, faculty preference, employment status, income levels, and the strength of religious practices. These findings highlight the crucial influence that socio-demographic and cultural factors have on shaping the decision-making path in the educational field. A detailed examination of the data points to significant differences in the level of individualism, with higher values measured among Muslim participants than among their Jewish counterparts, as well as similar patterns of risk aversion among both groups. These insights align with the observations presented in the studies of Gross and Gamal [10], who examined the tension between preserving a unique identity and integrating into Israeli society among Arab students and pointing to its long-term implications for their academic and professional paths.

The use of two complementary research designs, combining advanced tools for data collection and analysis, enabled a profound exposure to the complex dynamics guiding Jewish and Muslim students in choosing their field of study while emphasizing the points of difference and similarity in the influence of cultural, economic, and social factors. The first study yielded innovative insights into the increased tendency of Muslim students to be influenced by recommendations from teachers and educators, in contrast to their Jewish counterparts, who place more decisive weight on considerations of future earning potential. These findings fit well with the insights of Sharabi [6], pointing to the existing gaps in the perceptions and occupational motivations of Jewish and Arab university graduates. Furthermore, the research illuminated the differences between the two groups in their approach to the economic burden of higher education and the importance of acquiring early work experience, with Muslim students exhibiting heightened sensitivity to both aspects. The second study, which relies on an innovative use of the Conjoint Analysis method, added a significant layer to understanding the relative importance of various influencing factors and further highlighted the need to formulate educational policies and pedagogical practices tailored to the changing socio-cultural reality and economic constraints. This perspective receives significant reinforcement in the study of Lev Ari and Mula [9], documenting the contribution of intercultural interaction in teacher training to implementing multicultural approaches and cultivating inclusive capacity in the education system. An integrative comparison of the findings emerging from the two studies establishes the need to develop a holistic and multidimensional approach to planning education systems and curricula, considering the diverse dynamics influencing students from different socio-cultural backgrounds. Implementing these insights into decision-making processes can contribute to promoting inclusive and supportive educational environments tailored to the full range of needs and aspirations of all students.

The methodological and theoretical frameworks employed in this study align particularly well with both Wikan’s [36] concept of resonance and Appadurai’s [35] understanding of cultural modernity. Our findings demonstrate how Muslim students navigate what Appadurai terms ’the work of the imagination’ in crafting educational choices that embrace modern institutions while maintaining cultural distinctiveness. This dynamic exemplifies Appadurai’s observation on how local actors negotiate global cultural flows through their own cultural lenses. Similarly, Wikan’s emphasis on looking beyond surface differences to engage with shared human experiences proved invaluable in understanding how students from different backgrounds make meaningful educational choices within the same institutional framework. The higher levels of individualism observed among Muslim students, coupled with their maintained connection to collective cultural ties, illustrate what Wikan describes as the capacity for cultural understanding through resonance—where seemingly contradictory behaviors become comprehensible when viewed through the lens of shared human aspirations and challenges. These theoretical perspectives help explain how students can maintain distinct cultural identities while participating in shared modern educational institutions, making choices that sometimes transcend traditional cultural boundaries while remaining grounded in their respective cultural contexts.

In summary, the current research represents an important step in understanding Israeli society’s complex factors underlying educational choices. It offers a comprehensive and up-to-date perspective on the diverse cultural, social, and economic influences shaping the academic paths of Jewish and Muslim students. The findings emphasize the need to create an inclusive educational space that values diversity and grows out of recognition of the unique needs of each individual. However, it is worth noting that the research focused on the two main groups that make up the social mosaic in Israel. Thus, the full ethno-cultural complexity of the local academic system was not exhausted. Follow-up studies will be able to expand the scope and examine additional groups and trace the delicate mechanisms through which diverse identities are organized and cast side by side in the Israeli social fabric. At the same time, there is a need for longitudinal studies that track the changing influences on academic choices over time and provide a dynamic view of the interaction between social and personal forces throughout students’ life paths.

One notable limitation of this study is the relatively small sample size. Although the sample provided valuable insights into the factors influencing educational choices among Jewish and Muslim students, it may not fully represent the broader populations of these groups in Israel. The limited sample size could affect the generalizability of the findings, as it may not capture the full diversity of experiences and perspectives within these communities. Future research involving larger and more diverse samples is recommended to validate and extend the conclusions drawn from this study, ensuring a more comprehensive understanding of educational decision-making processes in multicultural societies. Furthermore, future research will examine the long-term effects of different choice patterns in the higher education system on economic and social mobility, emphasizing how academic paths may perpetuate or disrupt power structures and social stratification in Israeli society. This conceptualization will contribute to a more comprehensive understanding of the role of higher education in shaping society and promoting equal opportunities.

## Supporting information

S1 Appendix (DOCX)
