# Supplementary material for: Cultural, social, and economic influences on academic field choice among Jewish and Muslim students
Source: PLoS One. 2024 Dec 6;19(12):e0315276. doi: 10.1371/journal.pone.0315276 (PMC11623797; doi:10.1371/journal.pone.0315276)
Supplement: S1 Appendix — (DOCX) [file pone.0315276.s001.docx]

**Appendix**

This appendix contains the full list of items used in the individualism and risk aversion scales administered in the study.

Individualism Scale

Instructions: Please rate each of the following statements on a scale from 1 (Strongly Disagree) to 5 (Strongly Agree).

1. If the group is slowing me down, it is better to leave it and work alone.
2. To be superior, a person must stand alone.
3. Winning is everything.
4. Only those who depend on themselves get ahead in life.
5. If you want something done right, you've got to do it yourself.
6. What happens to me is my own doing.
7. I feel winning is important in both work and games.
8. Success is the most important thing in life.
9. It annoys me when other people perform better than I do.
10. Doing your best isn't enough; it is important to win.
11. Cooperating with someone whose ability is lower than one's own is less desirable than doing the task alone.
12. In the long run, the only person you can count on is yourself.

Risk Aversion Scale

Instructions: Please rate each of the following statements on a scale from 1 (Strongly Disagree) to 6 (Strongly Agree).

1. I enjoy taking risks.
2. I avoid situations of uncertainty.
3. I am not troubled by taking risks if my actions might yield substantial gains.
4. I consider the possibility of not taking risks as a main factor in my life.
5. People say that I enjoy taking risks.
6. I will take risks only very occasionally, if at all, if there is another alternative.
